# New Insights on Heat Shock Proteins as Regulators of Reactive Oxygen Species Across Various Stressors in Diseases

**DOI:** 10.1002/cbf.70173

**Published:** 2026-01-26

**Authors:** Paka Sravan Kumar, Triveni Kodi, Adarsh Gopinathan, Bharath Harohalli Byregowda, Krishnadas Nandakumar, Anoop Kishore

**Affiliations:** ^1^ Department of Pharmacology Manipal College of Pharmaceutical Sciences, Manipal Academy of Higher Education Manipal India

**Keywords:** external stress, heat shock proteins (HSPs), internal stress, oxidative stress, reactive oxygen species (ROS), stress

## Abstract

Living beings are persistently challenged by stress. Stress can be induced by internal stressors and external stressors. External stressors, including radiation, heat, heavy metals, nutritional imbalances, infections, and psychological stress, can induce protein denaturation, leading to misfolded or aggregated proteins. These stressors often cause overproduction of reactive oxygen species (ROS), leading to oxidative stress following inflammation. This cascade causes and accelerates various disorders, such as diabetes, neurodegenerative, respiratory, cardiovascular, autoimmune. This disease, in turn, becomes internal stressors, perpetuating cellular dysfunction through sustained ROS production and chronic inflammation, creating a self‐amplifying cycle that can lead to degenerative outcomes and organ failure. To cope with these stressors, cells initiate defense and protective mechanisms like antioxidant and heat shock proteins (HSPs). However, HSPs rapidly work to correct protein misfolding, mitigate oxidative damage, and reduce inflammation in response to external and internal stressors. HSPs increase the cell's efficiency to lower ROS levels and maintain the redox balance. On the other hand, cellular antioxidant regulatory role of HSPs includes suppressing apoptosis, modulating inflammatory signaling pathways (such as NF‐κB, MAPK, JAK‐SAT), inflammatory mediators (including TNF‐α, IL‐1β, IL‐6) and maintaining proteostasis, Therefore, HSPs play a significant role in cellular survival and function under stress. However, targeting HSPs represents a promising future strategy for managing stress related conditions with a variety of diseases.

## Summary

Reactive oxygen species, byproducts of cellular metabolism, are normally balanced by the cell's antioxidant defense system through redox homeostasis. Under stress (external and internal), excessive reactive oxygen species production disrupts redox homeostasis, leading to oxidative stress, inflammation, and cellular damage to DNA, lipids, and proteins. This damage triggers a vicious loop that increases reactive oxygen species levels. To counteract this, cells activate stress response pathways, including heat shock proteins, which play a vital role in protecting cellular components. Heat shock proteins stabilize damaged proteins, enhance antioxidant activity, and, when damage is irreversible, promote apoptosis to eliminate compromised cells, thereby protecting the integrity of cells and tissue under stress. Therefore, heat shock proteins are being explored as possible targets for reducing the negative effects in some disorders by increasing their levels.

## 1 Introduction

Stress is an internal or external stimuli or conditions such as extreme heat, cold, injury, etc., which influences physiological, biochemical, and molecular processes of organisms. These unfavorable stimuli are known as stressors. Based on the consequent effects on the organism, stress can be categorized as acute (transitory) or chronic (persistent). Acute stress (AS) is a transitory or short‐term stress that majorly occurs from challenging situations or immediate stressors. It leads to temporary physiological changes [1]. AS is mainly adaptive and protective in nature, since it activates defensive mechanisms to assist the organism cope with the stressor.

During an AS response, body activates protective mechanisms (such as behavioral, autonomic nervous system, neuroendocrine, immune, and biomolecular pathways) to cope with the stressors and undergo adaptation to balance the homeostasis [2]. Under AS, metabolic resources are utilized to deal with the heightened energy demands required for the fight‐or‐flight response. Blood glucose levels rise, lipids are released from adipose stores, and body temperature, heart rate, blood pressure, and respiratory rate increase to enable an effective response to the perceived threat. These physiological alterations are adaptive and support immediate survival, and the adaptive systems return to baseline once the stressor is no longer present [3]. Therefore, AS‐induced responses can be an adaptive physiological process that boosts immune protection after vaccination, injury and even certain types of cancers [4].

Chronic stress (CS) [5], occurs when the stressor continues over an extended period. Increased exposure to stress can result in cumulative and permanent physiological and psychological changes. Some of these changes can lead to increased risk of health problems, including cardiovascular disease, anxiety and depression etc [5]. CS may lead to dysfunctional physiological responses and is linked to various health conditions, including hypertension, metabolic syndrome, obesity Type II diabetes, and arthritis. For instance, deregulation of the circadian cortisol rhythm occurs concurrently as a detrimental consequence of CS [6]. Unlike AS, which does not significantly alter cellular gene expression, CS may also induce substantial alterations in the genetic expression.

Environmental stressors such as hypoxia, heavy metal pollution, UV radiation, starvation, and heat exposure can induce cellular stress (CS). For example, heat stress is particularly harmful because it leads to protein denaturation and functional loss in organisms. Elevated temperatures disrupt growth, development, reproduction, survival, and other physiological processes. With global warming, the intensity, frequency, and duration of heat stress events are increasing, and these conditions are predicted to intensify further [7]. These environmental stimuli are considered external stressors. External stressors primarily impair cellular function by inducing protein misfolding and elevating the production of reactive oxygen species (ROS), which are metabolic byproducts generated in the mitochondria, endoplasmic reticulum (ER), and lysosomes. At physiological levels, ROS act as signaling molecules that activate defense pathways. However, excessive ROS production leads to oxidative damage to lipids, proteins, and nucleic acids [8].

Chronic or persistent exposure to external stressors can elevate ROS levels to pathological levels, contributing to the development of metabolic disorders, immune dysfunction, neurodegenerative conditions, and respiratory diseases. Once established, these disorders themselves generate sustained oxidative stress and thereby function as internal stressors, further burdening cells and organ systems. To counteract such damage, the CS response activates protective systems, including antioxidant enzymes and heat shock proteins (HSPs). These defense mechanisms operate under both external and internal stress conditions, helping to restore redox balance, repair damaged proteins, and maintain cellular homeostasis. Both antioxidants and HSPs function independently and synergistically to reduce ROS levels and maintain cellular integrity. HSPs not only stabilize misfolded or denatured proteins but also regulate key signaling pathways involved in oxidative stress mitigation.

Major pathways modulated by HSPs include AKT, ERK1/2 (extracellular signal‐regulated kinase 1/2), MEK (mitogen‐activated protein kinase), p38 MAPK, Nrf2–NQO1, PGC‐1α (peroxisome proliferator‐activated receptor gamma coactivator‐1α), and NADPH oxidase (NOX) systems. Through modulation of these pathways, HSPs suppress excessive ROS accumulation, restore protein homeostasis, and enhance overall cellular resilience under stress [9, 10]. In this review, we discuss how external stressors trigger ROS generation, leading to cellular damage and disease, and how these resulting disorders function as internal stressors that further elevate oxidative burden. We also discuss how cells counteract ROS‐induced stress through antioxidant systems and HSPs, both individually and synergistically. Additionally, the review highlights the therapeutic advantages, therapeutic challenges, and both protective and pathogenic roles of HSPs, along with current HSP modulators and inhibitors, and future perspectives in HSP‐targeted interventions.

## 2 Cellular Response to Stress

CS involves disruptions in the internal environment, which can range from a minor change in the normal cellular processes to severe insults that can harm the structure or functioning of the cell. CS occurs from numerous stress factors such as injury hypoxia, hormones, inflammation, xenobiotics or higher concentrations of metabolites [11]. The stress factors can come from a variety of sources, both external and internal [12]. Extracellular stressors are considered extrinsic in nature, such as UV radiation, heat, pollution, xenobiotics, chemicals (such as heavy metals), diet (high protein or fat diet), alcohol etc., can adversely affect cellular function like protein denature, decreased metabolic activity, induce oxidative stress, organ damage, and DNA damage. Intracellular stressors involve factors such as inflammatory cytokines, autoimmune diseases, fat deposition, and neuroinflammation which disrupt the normal functioning of cells, and may lead to a variety of serious health problems like arthritis, heart disease, impaired immune function, insulin resistance, diabetes, and neurodegenerative disorders. Stressors can trigger a variety of stress responses and damage the intracellular macromolecules, including proteins, lipids, RNAs, and DNA [8]. For instance, Hypoxia, a CS condition characterized by oxygen deficiency and decreased ATP production, occurs when the body's oxygen demand exceeds its supply. This imbalance disrupts cellular organelles, leading to a metabolic crisis and disrupted homeostasis. During hypoxia, ROS are generated and damages cellular components, leading to pathological conditions [13] like inflammation, cardiovascular diseases, respiratory diseases, autoimmune diseases, and liver diseases. The CS response is a physiological mechanism in which a cell attempts to counteract a threat, cope with stressful circumstances and recover damage.

Eukaryotic cells have a natural ability to cope with variety of physiological stresses throughout their life cycle [12]. Certain CS response pathways or mechanisms have been highly conserved throughout evolution, such as the heat shock response and antioxidant defense systems against the ROS cycle [12, 14]. Survival CS response pathways and mechanisms protect the molecules of cells, cells and the organism from negative situations, it can also enhance apoptosis to eliminate the damaged or injured cells, when the survival strategies fail.

## 3 Cellular ROS Dynamics and Redox Reactions

ROS are highly reactive chemical entities generated through the partial reduction of molecular oxygen when they gain electrons., ROS include oxygen radicals and non‐radical derivatives [15, 16]. Superoxide anion (O₂⁻), hydroxyl radicals (OH), nitric oxide (NO), alkoxyl radicals (RO), thiyl radicals (RS), and lipid peroxyl radicals (LOO⁻) are the foremost examples of the radical ROS, whereas hydrogen peroxide (H_2_O_2)_, singlet oxygen (^1^O_2_), dinitrogen dioxide (N_2_O_2_), ozone (O_3_), hypochlorous acid are non‐ radicals ROS [17, 18].

### 3.1 Sources of ROS

A total of 90% of ROS are released by mitochondria by leaking the electrons during cellular respiration in the form of ATP under oxidative phosphorylation to produce energy. In addition to mitochondria, several other organelles such as peroxisomes, plasma membrane, cytosol, ER and nucleus and enzymes such as nicotinamide adenine dinucleotide phosphate oxidases (NOXs), xanthine oxidase (XO), cyclooxygenase (COX), and lipoxygenases (LOXs), forms ROS by functioning their role in the cell metabolism. These are the endogenous or internal sources or factors to form ROS. Transition metals ion such as zinc (Zn), iron (Fe), and copper (Cu), generate ROS through performing Fenton and Haber‐Weiss reactions. Studies also suggested that few toxic metals including vanadium, cobalt, chromium, arsenic, antimony, and nickel develop high concentration of ROS during redox reaction in eukaryotic cells. Miscellaneous sources such as exposure to radiation, ozone (O_3_), cigarette smoke, alcohol, air pollutants, pesticides, and industrial chemicals produce ROS. These are the exogenous or external sources or factors to form ROS.

### 3.2 Mechanism, Causes, and Effects of ROS in the Cell

Eukaryotic cells continuously produce endogenous ROS as a natural by‐product of cellular metabolism. At low levels, ROS play beneficial roles in regulating cell signaling, proliferation, differentiation, and programmed cell death. However, when ROS accumulate beyond physiological limits, they oxidize proteins, lipids, DNA, and causes oxidative stress in tissues [19], thereby contributing to the development and progression of numerous chronic and degenerative diseases [20, 21].

### 3.3 Maintenance of ROS Balance by Cells

The ROS is maintained by a balance between the enzymatic and non‐enzymatic methods. Enzymatic methods consist of activity of SOD (superoxide dismutase), TrxR1 (thioredoxin reductase‐1, CAT (catalase), GPx (glutathione peroxidase), NQO1 (quinone oxidoreductase‐1), HO‐1 (hemeoxygenase‐1), (Prxs) peroxidoxins, (Msr) methionine sulfoxide reductases [22, 23]. The non‐enzymatic methods involve molecules such as α‐lipoic acid (1,2‐dithione‐3pentanoic acid), polyphenols, melatonin, isoprenoids, NAC (N‐acetylcysteine), vitamin E, GSH (glutathione), and vitamin C for antioxidant defenses. Among the enzymatic defenses, GPx, CAT, and SOD can be considered as the first line of defense against ROS and oxidative stress. These enzymes work together to neutralize harmful ROS, preventing their detrimental effects [24].

ROS can be produced by both internal and external stressors. These highly reactive molecules play a main role in redox reactions. Generally, oxidative stress occurs when the ROS levels overcome the body's natural antioxidant defenses, causing an imbalance in redox homeostasis. Redox reactions are fundamental to CS responses, and protein phosphorylation and dephosphorylation play crucial roles in regulating cellular functions. These reactions, influenced by redox balance, cooperatively regulate a vast variety of biological functions [25]. ROS levels can be dependent on oxygen concentrations. ROS have historically been considered of harmful results of surviving in an aerobic condition since they are known to degrade cellular macromolecules, may cause cell death. Recently, studies have shown that ROS can act as signaling molecules, regulating number of cellular activities, including proliferation. ROS is considered a secondary messenger. It has been reported that ROS may operate as second messengers, activating MAP kinase (mitogen‐activated protein kinases), ion channels, tyrosine phosphatases, and tyrosine kinases to start signaling cascades. ROS can act as both toxic and secondary messenger functions. The fact that ROS can function as both toxins and signaling molecules may be due to variations in their concentrations, subcellular localization, and pulse length [26]. Elevated ROS levels may trigger the cell's heat shock response by directly activating HSF1 (heat shock factor 1), causing damage to cellular macromolecules, mitochondrial malfunction, and ER stress. HSP synthesis is a defensive reaction that aims to repair damaged proteins, inhibit protein aggregation, and keep cells functioning under stress [25].

### 3.4 Mechanism of Enzymatic and Non‐Enzymatic Antioxidants at Cellular Level

SOD is the key enzymatic antioxidant that neutralizes superoxide radicals by converting them into oxygen and hydrogen peroxide, which is then detoxified into water and oxygen by glutathione peroxidase and catalase. Other enzymatic antioxidant systems including glutathione, thioredoxin (via TrxR1), HO‐1 (through bilirubin production), NQO1, peroxiredoxins, and methionine sulfoxide reductase further neutralize ROS or repair oxidative damage. Non‐enzymatic antioxidants such as α‐lipoic acid, polyphenols, melatonin, isoprenoids, NAC, vitamins C and E, glutathione, and flavonoids complement these defenses by donating electrons or hydrogen atoms to stabilize free radicals. Overall, these enzymatic and non‐enzymatic mechanisms work together to maintain cellular redox balance [27, 28, 29, 30, 31, 32, 33, 34, 35] (Table 1).

**Table 1:** Roles and mechanisms of antioxidants.

Antioxidant	Type and role	Mechanism in redox reaction
Superoxide dismutase (SOD)	Enzymatic/Catalyst	Metabolizes superoxide into H_2_O_2_ and O_2_ thereby mitigating oxidative stress) [27].
Catalase (CAT)	Enzymatic/Catalyst	Neutralizes the oxidant H₂O₂ by converting it to H₂O and O₂) [27].
Glutathione peroxidase (GPx)	Enzymatic/Catalyst	Reduces H₂O₂ and organic hydroperoxides (ROOH) to H_2_O [28].
Thioredoxin reductase‐1 (TrxR1)	Enzymatic/Catalyst	Catalyzes the reduction of thioredoxin which neutralizes H_2_O_2_ [29].
Hemeoxygenase‐1 (HO‐1)	Enzymatic/Catalyst	Breaks the ROS include, peroxyl, radicals, O₂⁻ [30].
Quinone oxidoreductase‐1 (NQO1)	Enzymatic/Catalyst	Breaks the ROS include H_2_O_2_, peroxyl, radicals, O₂⁻ [31].
Peroxidoxins (Prxs)	Enzymatic/Catalyst	Neutralize H_2_O_2_ and organic hydroperoxide (ROOH) [32].
Methionine sulfoxide reductases (Msr)	Enzymatic/Catalyst	Reduces ROS including H_2_O_2_, hydroxyl radicals [33].
Glutathione (GSH)	Non‐enzymatic/Reducing agent	Neutralizes a hydroxyl radical, and the thiol group (‐SH) of glutathione donates a hydrogen atom (H) to the radical, effectively neutralizing it [34].
Thioredoxin (Trx)	Non‐enzymatic/Reducing agent	Breaks the ROS including H_2_O_2_, and hydroxyl radicals [29].
*N*‐acetylcysteine (NAC)	Non‐enzymatic/Reducing agent	Breaks the ROS including H_2_O_2,_ and hydroxyl radicals [35].
Melatonin	Non‐enzymatic/Reducing agent	Breaks the ROS including H_2_O_2_, and hydroxyl radicals [35].
Vitamin C	Non‐enzymatic/Reducing agent	Encounters a free radical (, ascorbic acid donates one of its electrons, resulting in the formation of a stable product [34].
Vitamin E	Non‐enzymatic/Reducing agent	Reduces free radicals by donating a hydrogen atom (H) from its hydroxyl group (‐OH) in the chromanol ring. This reaction converts the free radical into a stable molecule [34].
Polyphenols (e.g., Flavonoids)	Non‐enzymatic/Reducing agent	Reduce free radicals by donating a hydrogen atom (H) from its hydroxyl group (‐OH) in the chromanol ring. This reaction converts the free radical into a stable molecule [33, 34, 35, 36]
Isoprenoids (e.g., Carotenoids)	Non‐enzymatic/Reducing agent	Reduce free radicals by donating a hydrogen atom (H) from its hydroxyl group (‐OH) in the chromanol ring. This reaction converts the free radical into a stable molecule [33, 34, 35, 36].
α‐Lipophilic acid	Reducing agent/Reducing agent	Breaks down ROS including H_2_O_2_, hydroxyl radicals [35].

Disturbances in redox balance activate several proteins and peptides with antioxidant functions, including HSPs. Under stress, HSP27, HSP60, and HSP70 enhance key antioxidant enzymes such as SOD, CAT, and GST [7]. In ischemia, elevated HSP70 supports recovery of the glutathione‐dependent thiol–disulfide system, and exogenous HSP70 strengthens glutathione activity in neurons. Melatonin further increases HSP70 expression through MT1/MT2 receptor signaling, inhibition of NF‐κB (nuclear factor‐kappa B) nuclear translocation, and suppression of iNOS and proinflammatory cytokines. As a potent antioxidant, melatonin decreases free radicals and limits DNA damage [9]. HSP90 also exhibits antioxidant properties by binding oxidized phospholipids and scavenging radicals such as DPPH [37]. Some antioxidants, including glutathione, can directly modulate HSP expression and activity, highlighting reciprocal regulation between Hsps and antioxidant systems [38, 39].

## 4 HSPs: Classification, HSF‐1 Regulation, and CS Protection

HSPs are induced by various stressors, including infections, heavy metals hypoxia, ethanol exposure ischemia etc., and are upregulated in numerous diseases such as autoimmune, metabolic, neurodegenerative, respiratory, and inflammatory conditions [40, 41]. HSPs can confer protection against severe types of potentially lethal stress, by a phenomenon known as preconditioning, observed across various cell types and tissues. Beyond their protective roles, HSPs contribute to cellular development and hemostasis. A large number of stress‐induced proteins are molecular chaperons, necessary for the transport, biosynthesis, folding of proteins [42, 43]. HSPs are structurally, functionally, and evolutionarily highly conserved proteins and have a very important role in the maintenance of cellular proteostasis. They function as molecular chaperones, utilizing housekeeping and chaperone mechanisms to ensure cellular stability under various conditions, including inflammation, immune responses, ion channel repair, and more.

**Figure 1:**
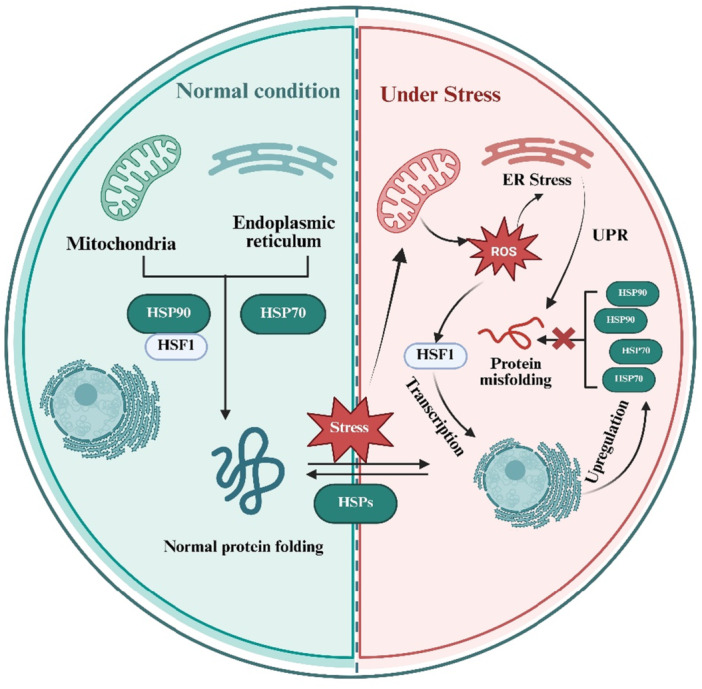
Schematic illustration of HSPs role, activation, and mechanism in normal and stressed cells: In normal cells, and under normal condition, HSF1 is bound to the HSP90 in the cytoplasm and keeps it inactive. HSP70 and HSP90 in normal healthy cell facilities proper protein folding. During stress, ROS activate the UPR (unfolded protein response), leading to protein misfolding. The misfolded proteins compete for HSP90, releasing HSF which initiate transcription then HSPs levels are increased to counteract ROS‐induce damage by refolding the misfolding proteins and maintaining cellular homeostasis. Abbreviations used: ER stress, HSF1, HSP90, HSP70, ROS, UPR.

The main characteristic of HSPs is their heightened expression during stress. This assists in encountering and protecting cells from several stressors, such as high temperatures, exposure to poisons (heavy metals, ethanol) inflammatory responses, tissue hypoxia, UV radiation, etc. Under stress, HSPs become the cell's first line of defense, performing essential functions like: newly synthesized polypeptides folding, degrading of misfolded proteins, refolding unstable proteins, and dissociation of protein aggregates. HSPs can be categorized based on their molecular weight [44, 45]. Large molecular weight species include HSP40, HSP60, HSP70, and HSP90, the smaller molecular weight include HSP32, HSP27, HSP22, and HSP20. Small HSPs are the initial line of defense against stress, defending against oxidative harm, apoptosis and cytoskeletal instability [46, 47, 48], while large HSPs are more active in protein quality control and signaling regulation. They work together to establish a coordinated chaperone network, with small HSPs stabilizing damaged proteins and large HSPs refolding them [49, 50]. The heat shock response's primary transcriptional regulator is HSF1 (Figure 1). In response to a variety of stressors, including heavy metals, heat shock, oxidants, and proteotoxic chemicals, HSF1 binds to the promoters of HSP genes and coordinate their expression [51]. The activation cycle of HSF1 is a complex procedure with numerous phases that are highly regulated. Under normal circumstances, HSF1 resides primarily in an inactive monomeric form. When HSF1 is exposed to stress, it undergoes a series of modifications, leading to its conversion into a DNA‐binding‐competent, active trimeric form. The trimeric form of HSF1 binds to the heat shock elements (HSEs) located in the promoters of HSPs genes, thereby initiating their transcription and ultimately leading to the production of HSPs. The activation of HSF1 causes its nuclear accumulation in response to a bipartite nuclear localization signal. The mechanisms that keep HSF1 in its inactive monomeric state. HSF1 activation occurs through distinct pathways depending on the stressor. As well as studies have suggested that the increased temperature induces conformational abnormalities in HSF1, which causes oligomerization and activation [52].

## 5 Crosstalk Between the HSP and ROS During Stress

Increased ROS causes oxidative stress, which can oxidize proteins and activate HSF1. At the same time, the body's antioxidant defense system both enzymatic and non‐enzymatic gets activated to counter ROS. This antioxidant oxidant defense system can activate HSF1 to modulate the antioxidants levels in deficiency and excess through HSPs [53]. According to Klumpen et al., ROS is the primary mediator of early HSP development, whereas protein damage and HSF1 are the main causes of later HSP expression. ROS indirectly activate HSF1 by oxidizing protein thiols, which triggers the Keap1/Nrf2 and HSP90/HSF1 signaling pathways. Activation of either HSF1 or Nrf2 shifts the cellular redox balance toward a more reduced state.

**Figure 2:**
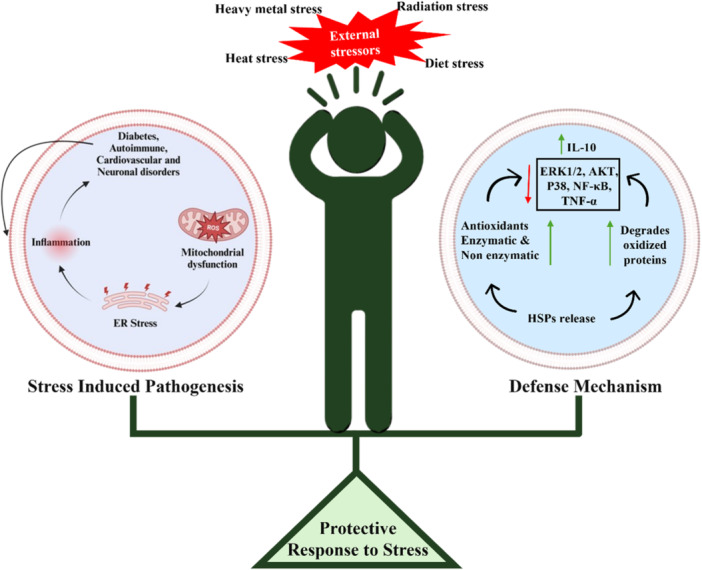
Schematic illustration of how HSPs counteract external and internal stressors in the cell. The external stressors such as heavy metals, radiation, heat, diet cause mitochondrial dysfunction and ER stress which increases ROS. This ROS induces inflammation which triggers various conditions such as diabetes, autoimmune, cardiovascular, and neuronal disorders. However, the cell activates defense mechanisms against stress including HSPs, antioxidants and proteasomal activity which aids in reduction of oxidative stress, degradation of proteins, and regulation of inflammatory pathways. Maintaining the equilibrium between stress (external and internal) induces pathogenesis and defense mechanisms results in protective response. Abbreviation used: ER stress, HSPs, IL‐10, ERK1/2, AKT, P38, NF‐κB, TNF‐α.

Antioxidant system stabilizes elevated ROS levels via both enzymatic and nonenzymatic mechanisms. HSPs are multipurpose proteins that have close connections with the antioxidant defense systems (Figure 2). Hence, HSPs protect cells by controlling ROS levels. HSP regulates ROS levels in several ways. The effect of HSPs on regulation of ROS levels can be explained in terms of maintaining cellular homeostasis [54]. HSPs have been found to be linked to antioxidant systems to mitigate ROS levels in cells [55]. For instance, HSP70 upregulates free 20S proteasome, which enhances the ability to breakdown oxidation and aggregation of proteins [56].

HSPs can also prevent apoptotic cascades by ROS. The AKT and ERK1/2 (Extracellular signal—regulated kinase1/2) pathways are significantly activated in response to oxidative stress [57]. During an increase in oxidant (H_2_O_2_) levels, the levels of Akt, ERK1/2 are found to be elevated [58]. However, AKT interacts with MEK (Mitogen—activated protein kinase) and ERK pathways [59]. The complex of AKT‐HSPs formed stabilizes AKT, which helps to keep the cells protected from cell death [60]. HSP70 and HSP90 bind to NADPH Oxidase (NOX) proteins specifically to control the generation of ROS, and they help stabilize the enzymes. Production of ROS generated by NOX can be effectively suppressed by HSP70 alone. HSP70 proteins possessing reactive cysteine residues have been discovered to be undergoing cysteine alterations, suggesting that they can effectively sense redox alter and contribute to the control of redox homeostasis [61]. HSP70 protects pulmonary endothelial cells by mitigating bacterial toxin‐induced mitochondrial oxidative stress, which is a vital factor in compromising endothelial barrier function [62]. HSP22 is upregulated in AECs (Airway Epithelial Cells) in response to oxidative stress, particularly after ozone exposure. This induction shields AECs from oxidative damage by activating the Nrf2‐NQO1 pathway of nuclear factor erythroid 2‐related factor 2‐NAD(P)H [63]. Similarly, oxidative stress‐induced HSP20 in AECs enhances Nrf2 translocation, leading to increased NQO1 expression. HSP20 itself functions as an antioxidant [64]. Oxidative stress triggering the ROS generation are more prevalent in hypoxic conditions [65].

Hypoxia triggers oxidative stress and the production ROS which causes altered protein synthesis, mitochondrial malfunction, and an imbalance in energy, lipid, and carbon metabolism. On the other hand, hypoxia can lead to a loss of protein integrity through disturbances in the redox balance, resulting in protein structural degradation, mostly among mitochondrial proteins [66]. To counteract these negative consequences, HSPs can be activated to preserve proteostasis in hypoxic circumstances [67]. Therefore, HSPs possess a wide role in controlling ROS levels in a variety of cellular pathways and conditions.

## 6 HSPs and ROS Coordinated Response in Various External Stressors

### 6.1 Heat Induced Stress

High temperature (heat) affects metabolism and increases the metabolic heat production in living beings. Consequently, this triggers behavioral and physiological mechanisms that attempt to decrease metabolic heat production and enhance heat dissipation. When these mechanisms fail, an imbalance develops between the amount of heat absorbed or created and the amount of heat lost to the environment, resulting in heat stress. When organisms sense heat stress, their cells' metabolic rate rise and they generate excessive ROS, including hydrogen peroxide (H_2_O_2_) and singlet oxygen. Excess ROS can react with membrane lipids, generating MDA (malondialdehyde). However, when ROS synthesis surpasses the body's processing capabilities, cells undergo oxidative stress, leading biomolecules like lipids, proteins, and DNA to be destroyed [68].

Heat stress directly impacts the organisms health by generating oxidative stress and immunological suppression, which may lead to death [69]. It is found that heat stress decreased the levels of IL‐10 and increased the generation of TNF‐α in rats, suggesting that HS may cause an inflammatory response. A taxonomic and functional differences between the normal and heat‐stressed faeces microbiome groups is also observed. Hyperthermia disturbs the mechanism of ROS production and antioxidant levels, which causes oxidative stress [70]. The alterations between ROS generation and the body's antioxidant defenses promote HSP formation [16]. ROS generation is produced by several stresses, including oxidative stress, heavy metal stress, and hyperthermia. Heat shock‐induced HSP generation in Human Primary Dermal Microvascular Cells (hPDMCs) reveal that hyperthermia promotes HSP production by generating ROS expression and activating p38/protein kinase B (Akt) signaling, therefore improving HSF1 transcription activity, which contributes to HSP production. Akt and p38 MAPK (p38 mitogen‐activated protein kinase) signal pathways are needed for heat shock response in many cells [25].

Hyperthermia‐induced imbalance between ROS generation and antioxidant levels may generate oxidative stress, which leads to DNA damage [70]. Even without heat shock, oxidative stress may activate HSF1 on its own [70]. Temperature controls the reproductive activity of the Testis in animals. A rise in testis temperature has a deleterious effect on spermatogenesis. In animals, the testis temperature is usually 2°C–8°C lower than the whole‐body temperature. Testicular heat stress causes oxidative damage, which reduces testosterone levels in circulation and slows the proliferation of testicular cells. Heat‐induced PGC‐1α (Peroxisome Proliferator‐Activated Receptor Gamma) levels and localization in the Testis of mice, together with enhanced PGC‐1α expression in germ cells and Leydig cells, may form a counter‐reaction to oxidative stress, along with HSP70 [71]. Hsp27, Hsp70, and Hsp90 play key regulatory roles in heat stress by stabilizing protein conformation or aiding in protein aggregation and dispersion by inhibiting ROS and through upregulation of Hsp27, Hsp70, and Hsp90 [72].

### 6.2 Stress Due to Diet

The quality, quantity, composition, and timing of food intake significantly influence human health by affecting nutrient availability [73]. Dietary choice leads to the development of pathologic conditions like inflammation, hypertension, hypercholesterolemia, obesity, these promotes the risk of interrelated disorders like cardiovascular diseases as well as metabolic disorders (Ex. Type 2 diabetes). Nutrition and diet are crucial, regulating factors in both the prevention and management of metabolic diseases [74, 75, 76, 77]. Unhealthy dietary habits are linked to an accelerated rate of metabolic disorders, which can impose a significant economic burden on healthcare systems worldwide [78]. Most people consume foods high in saturated fat and carbohydrates [79]. Such dietary composites are considered to rise ROS levels enough to trigger relevant transcription factor, such as the antioxidant response element‐regulated response and/or the heat shock response, but not cell death signaling pathways [79, 80]. Malnutrition causes mitochondrial oxidative metabolism, which increases ROS generation and causes an imbalance between oxidative stress induction and redox, these both contribute inflammation. Abnormal generations of ROS and proinflammatory cytokines occur, that are produced in hypertrophic adipocytes as a result of unhealthy diet [81].

### 6.3 Stress Due to High‐Fat Diet (HFD)

Diets rich in dietary fibers, high in nutrients and monounsaturated fatty acids, have currently been replaced by diets high in refined sugars, and saturated fats. High intake of fatty acids regulates the activity of various transcription factors, initiating downstream signaling cascades. PPARs (Peroxisome proliferator‐activated receptors) are ligand‐activated transcription factors that play a central role in sensing and regulating lipid metabolism. As well as Polyunsaturated fatty acids react with sterol regulatory element binding proteins and other transcription factors in the liver, influencing gene expression related to their synthesis and promoting the uptake of phospholipids, fatty acids, and cholesterol [82]. HFDs have been shown to induce cellular and molecular damage, triggering an oxidative stress response. This leads to disproportion between free radicals of the ROS and stabilizing antioxidant enzymes in the body. The resulting oxidative stress activates signaling pathways involving HSPs and MAPKs to protect against oxidative damage. However, this process may also lead to lipid peroxidation, protein modification, and insulin resistance.

Antioxidants and HSPs typically function by scavenging and neutralizing free radicals, thereby preventing oxidative damage. However, when antioxidant levels are insufficient, oxidative stress leads to lipid peroxidation, protein misfolding, DNA damage, and other negative consequences [83, 84, 85] Furthermore, Antioxidant proteins Bcl‐2 and HSP70 exert beneficial effects by suppressing ICAM‐1 (Intercellular Adhesion Molecule 1) expression and decreasing leukocyte and platelet adhesion. Together, these changes protect vascular endothelial cells, reduce inflammation, and FeCl_3_‐induced thrombus formation in HFD‐fed hamsters [86].

### 6.4 Stress Due to Alcohol

Alcohol is primarily metabolized through alcohol dehydrogenase pathway, followed by the microsomal ethanol oxidizing system [87]. The alcohol dehydrogenase pathway, predominantly occurring in liver cytosol, utilizes alcohol dehydrogenase enzymes to breakdown ethanol. microsomal ethanol oxidizing system, involving enzymes like catalase and CYP2E1 (the enzyme belonging to the cytochrome P450 family) located in peroxisomes and microsomes, respectively, is a secondary pathway. Both pathways are oxidative. While a non‐oxidative pathway exists, producing phosphatidic acid (PA) and fatty acid ethyl esters, it is less significant [88]. At low blood alcohol concentrations, alcohol dehydrogenase is the primary metabolic enzyme. As alcohol levels rise, Microsomal ethanol oxidizing becomes increasingly involved in ethanol breakdown [89]. NAD^+^/NADH (Nicotinamide adenine dinucleotide and nicotinamide adenine dinucleotide) electron‐rich (reduced) ratio plays an imperative role in cellular redox balance. Response Protein 1 regulated by contributors like Sterol Regulatory Element Binding Protein 1c and Early Growth Response Protein 1, contributes to ethanol metabolism and can impact the NAD+/NADH ratio.

The microsomal ethanol oxidizing system pathway is another important system involved in ethanol oxidation. Understanding these factors is essential for understanding the metabolism of ethanol and its potential effects on cellular function. Alcohol consumption may lead to ROS production, which damages tissues and cells. These ROS can, in turn, trigger the production of pro‐inflammatory cytokines, like TNF‐α. Pathologies interplay between alcohol, ROS, and TNF‐α shows a significant role in various alcohol‐related pathologies. Numerous mechanisms for alcohol‐induced modulation of TNF‐α production by macrophages have been shown in studies. These include changes in NF‐κB activity, a rise in ROS production, an increase in Erk1/2 activity to promote TNF‐α transcription, an increase in p38 MAPK activity that leads to an increase in TNF‐α mRNA stability, and modifications in the TLR4‐CD14 Toll‐like receptor 4 and CD14 (Cluster of differentiation 14) receptor complex and downstream signaling molecules [90, 91].

Ethanol exposure, whether acute or chronic, can induce stress responses in the body, including HSFs activation and an upregulation in HSP levels. This response is similar to that seen with other stressors. The liver and brain are particularly sensitive to ethanol‐induced stress, and increased HSP expression has been observed in these tissues. Studies in rats have shown that ethanol exposure can lead to elevated levels of HSP70, a protein associated with liver injury. Additionally, in mice, short‐term ethanol administration has been found to increase the protein and mRNA levels of HSP72 and HSF1 protein in the liver [92]. HSPs can play a crucial role in maintaining the structure and function of the cytoskeleton, a cellular component that is particularly vulnerable to ethanol‐induced stress.

Chaperones, a class of HSPs, mediates the assembly of cytoskeletal components and provides protection from damage during stress conditions. For example, studies have shown that the overexpression of HSP70 or αB‐crystallin (small HSP) can help preserve the integrity of microtubules in rat neonatal cardiac myocytes subjected to simulated ischemia. Additionally, both αA (small HSP) and αB‐crystallin have been found to inhibit the depolymerization of actin filaments induced by cytochalasin D and the heat‐induced aggregation of actin filaments [93]. HSP27 overexpression provides protection from actin fragmentation that is caused by oxidative stress. This stability of microfilaments relates to a higher cell survival rate after oxidative stress exposure. HSPs and their regulator, HSF‐1, can influence the regulation of TNF‐α, a pro‐inflammatory cytokine. In acute alcohol exposure, HSF‐1‐induced HSP70 may play a role in inhibiting inflammation. However, HSP90 has been implicated in the induction of pro‐inflammatory cytokines in alcoholic liver injury [94].

### 6.5 Stress Due to Heavy Metals

Heavy metals in the environment, including lead, mercury, cadmium (Cd), copper, nickel, arsenic, and (exceptionally) aluminum, pose significant health risks due to their high density and the body's inability to effectively eliminate them. These metals can enter our bodies through various ways, such as contaminated air, water, construction materials, cookware, and even clothing. Unlike essential minerals, heavy metals accumulate in vital organs like the bones, liver, kidney, and brain, deranging their normal functions. Their toxicity varies, with some causing harm through inhalation while others pose risks upon ingestion. Moreover, they can replace essential elements in tissues, impairing cellular processes [95]. The persistent accumulation of these non‐metabolizable substances can lead to severe health consequences.

Understanding the specific dangers of each heavy metal is crucial for effective prevention and mitigation strategies. Heavy metals, ROS, and HSPs are all interrelated. Heavy metals enhance ROS production, causing cellular damage. HSPs are then activated to assist cells resist this harm [96, 97]. However, Heavy metals can raise ROS, which accelerates oxidative degradation of proteins, lipids, and DNA in several organs such as the kidneys, lungs, testis, heart, liver and bones [24]. HSP families can function particularly to detect certain heavy metal intoxications. HSP27 can reduce, neutralize, or avoid Cd's cellular cytotoxicity.

HSP27 is a multifaceted protein acting as molecular chaperon, antioxidant, anti‐apoptotic protein and plays a significant role in actin cytoskeleton remodeling. HSP27 plays a cytoprotective role by diminishing the ROS level and elevating the GSH level during oxidative stress. HSP27 increases the GSH level which is associated with increased glucose‐6‐phosphate dehydrogenase (G6PD) activity. Sanjay Saini et, al. study provided evidence that Cd‐ exposure decreases the GSH level in the MTs of Cd‐exposed Drosophila larvae. GSH level was investigated after the modulation of hsp27 in Malpighian tubules. A significant increase in the level of GSH was observed in Malpighian tubules. However, the protective role of hsp27 overexpression in safeguarding against Cd‐induced renal toxicity in melanogaster. By overexpressing hsp27, the occurrence of cell death in the Malpighian tubules of Cd‐exposed larvae was rescued [98]. Overexpression of HSP27 in cells can be used as a biomarker since it protects cells from oxidative stress and apoptosis and makes them resistant to Cd exposure [96, 99]. Bioflavonoids, like taxifolin, were employed to stimulate HSP27 overexpressing, which decreased Cd ion poisoning by inhibiting apoptotic death processes [100]. HSP70 levels are very sensible to the attacks caused by the environmental factors and persuaders like heavy metals [101]. HSP70 elevated levels is an excellent self‐defense mechanism that provides protection against Cd ions exposure. stress can generate HSPs.

HSPs are implicated in the molecular process of manganese toxicity in cock livers as well as rats [102, 103]. In chicken livers, dietary MN supplementation increased the HSPs at all tested doses [103]. Cd enters the lungs via the airways, causing oxidative stress and cell death. Thus, HSP27 and HSP70 can assist in reducing Cd oxidative stress [104]. Thorium (Th) stress has been shown to raise intracellular ROS levels, indicating a possible involvement in DNA damage, mitochondrial membrane potential loss, and membrane lipid peroxidation. Among the several HSPs, HSP70, HSP90 were shown to be changed in response to Th‐dioxide and Th‐nitrate exposure [105].

### 6.6 Stress Due to Radiation

Radiation arises from both natural and artificial sources. Natural radiation sources include terrestrial radiation, internal radiation and cosmic radiation. Artificial sources include medical radiation, nuclear power plants, and industrial radiation. Cosmic radiation comes from outer space and includes the sun and other celestial bodies. This includes cosmic rays that interact with the Earth's atmosphere, resulting in secondary particles reaching the surface. Terrestrial radiation is released by naturally occurring radioactive elements such as uranium, thorium, and radon in the earth's crust. Internal radiation is caused by naturally occurring radioactive chemicals in the human body, such as potassium‐40 and carbon‐14 which are naturally present in the human body. Medical radiation is used in diagnosis and treatment, including X‐rays, CT scans, and radiation therapy. Nuclear radiation is produced as a byproduct of nuclear fission in nuclear reactors. Although well‐contained, tiny quantities may be discharged into the environment. Industrial radiation is utilized in industrial applications such as radiography for material inspection, medical equipment sterilization, and food irradiation [106, 107, 108]. Radio frequencies are currently being utilized in a variety of sectors, including networks, medicine, wireless communication devices, and even space missions [109]. The ubiquitous usage of 2.45 GHz radiation, in particular, is increasing, raising concerns about possible health hazards [110, 111]. 2.45 GHz radiofrequency (RF) is a negative external stressor that affects the activity and homeostasis of parafollicular cells in the rat thyroid gland [112]. UV‐B radiation exposure decreases the survival rate of T. japonicus in a dose‐dependent manner, inducing oxidative stress through the production of ROS. HSPs, (particularly HSP70, HSP90, and HSP20), antioxidants (GSH, GSH‐dependent enzymes, and SOD), and cell signaling proteins (p53 and p38) may protect against UV‐B‐induced damage by regulating DNA repair and apoptosis [113]. HSP104, a protein involved in regulating CS responses, also influences the development and magnitude of induced radiation resistance and thermotolerance [114].

Radiation‐induced fibrosis (RIF), which may occur in skin and subcutaneous tissue, lungs, gastrointestinal and genitourinary tracts, as well as any other organs in the treatment field. Radiation injury triggers inflammation and ultimately stimulates trans differentiation of fibroblasts into myofibroblasts. In addition to their excessive proliferation, these myofibroblasts produce excess collagen and other extracellular matrix (ECM) components, which is compounded by a reduction in remodeling enzymes. Subsequent fibrosis reduces tissue compliance and in many cancer patients and particularly those with head and neck cancer cause cosmetic and functional impairment that significantly impact quality of life. HSP70 is associated with organ and tissue fibrosis, such as renal fibrosis, myocardial fibrosis, peritoneal fibrosis, liver fibrosis, and pancreatic fibrosis. Additionally, HSP70 has an antagonistic effect on EMT, which participates in the process of fibrosis. More recently, it has been demonstrated that HSP70 interacts with vascular smooth muscle cells, a major producer of ECM protein, through membrane‐bound TLR4 to promote ECM production [115, 116]. Downregulation of HSP90ab1 is likely to exacerbate cellular damage in radiation‐exposed tissues.

The role of HSP90 in RIF suggests that distinct regulatory mechanisms may be involved in fibrosis caused by mechanical damage versus radiation‐mediated damage [117]. HSP70 and HSP47 expression after laser irradiation using different laser configurations have been analyzed to assess their potential role in CS responses. The impacts of laser stimulation were evaluated immediately upon irradiation and during healing process [118]. Radiation treatment induces the generation of ROS, as well as cell growth and differentiation. Ionizing irradiation, even at sublethal levels (less than 5 Gy (A Dose of Ionizing Radiation), causes HSP70 production in the cytosol of normal cells. Irradiation causes cells to produce ROS, which triggers the creation of HSP70. Elevated intracellular HSP70 levels are shown to play an essential role in stress recovery [119]. HSP70 expression can be impacted by radio frequencies. 2.45 GHz altered HSP70, leading to increased stress and reduced anti‐inflammatory activity [111]. HSP70 keeps Akt phosphorylated (active), which inhibits the activation of apoptotic pathways and saves photoreceptor cells. HSP70 makes cells more resilient to stress by stabilizing Akt and preventing its dephosphorylation. Furthermore, HSP70 reduced H₂O₂‐caused apoptosis in epithelial cell (corneal) by inhibiting the activities of caspase‐3 and caspase‐9 in vitro. Exposure to UVB radiation generates excessive ROS in the cornea, triggering the activation of several cellular signaling pathways. This includes the stimulation of pro‐inflammatory mediators and NF‐κB, as well as direct damage to proteins and DNA. Geranylgeranylacetone (GGA) administration attenuated UVB‐induce corneal damage without changing ROS production. However, GGA increased HSP70 levels in the corneal tissue, which subsequently decreased NF‐κB activation both in vivo and in vitro, independent of ROS suppression [120].

## 7 Coordinated Response of HSPs and ROS During Various Intracellular Stressors

Inflammation has been considered as the primary defense stage of the intrinsic immune system, reacting to cellular injury caused by oxidative stress, ROS, xenobiotics, and other stressors. Chronic inflammation destroys cells and is a key factor in the progression of several inflammatory disorders [121]. such as obesity, neuro, diabetes, cardiovascular, autoimmune disorders. However, ROS causes severe inflammatory diseases. For instance, increased ROS levels in the airways have a key role in the development and evolution of hereditary inflammatory diseases (HIDs) by generating intense inflammation. Therefore, HSPs possess anti‐inflammatory properties by suppressing the NF‐κB, as well as by reducing the c‐Jun NH2‐terminal kinase pathway and caspases [122]. As well as HSPs inhibit inflammation via the prevention of pro‐inflammatory cytokines including tumor TNF‐α. On the other hand, reducing HSPs levels causes more severe inflammation, as proved in several HIDs. HSPs are highly expressed at inflamed areas, potentially aiding in the refolding of denatured proteins caused by ROS‐induced severe injuries [54].

### 7.1 Diabetes

Diabetes is a disorder involving both metabolic and inflammatory processes, primarily resulting from elevated blood sugar levels, known as hyperglycemia [123]. Hyperglycemia is triggered by various metabolic signaling pathways, that causes ROS generation, release of cytokines and leading to cell death, which leads to the progression of complication linked with diabetes. In diabetes, hyperglycemia activates mitochondrial respiratory chain enzymes such as peroxidase, cyclooxygenases, nitric oxide synthase, and xanthine oxidase leading to the production ROS [124].

Hyperglycemia induces ROS production and oxidative stress, which is linked to cellular damage in numerous organs and, eventually, contributes to the development of diabetic complications. Apparently, hyperglycemia induces oxidative stress and ROS, which contributes to cellular death through a variety of pathways and, eventually, tissue damage. In several studies, diabetes complications caused by hyperglycemia seem to be connected to an imbalance of ROS, which results in oxidative stress and cellular death [125]. As a result, inhibiting ROS formation may play a key role in diabetic complications management. However, in diabetes the high levels of acute or chronic glucose enhance ROS generation and activates TLR4 leading to apoptosis in the β‐cells. ROS of diabetes triggers ER stress, which causes apoptosis. In diabetic cardiomyocytes, apoptosis is triggered by a crucial ER stress sensor called protein kinase RNA (PKR)‐like endoplasmic reticulum kinase (PERK) [126, 127]. ROS, free fatty acids, and ER stress activate c‐Jun N‐terminal kinase (JNK) pathway, which leads to insulin resistance. JNK serine phosphorylates IRS‐1 (ser307) which is the proximal step in insulin signaling to disrupt IRS‐1. Thus, overproduction of HSP72 inhibits the apoptotic and ROS. HSP72 physically interacts with JNK1, preventing its phosphorylation by upstream kinase Stress‐Activated Protein Kinase 1 (SEK1), thereby suppressing the JNK1 signaling pathway. In mice, elevated HSP72 levels reduced NF‐κB p65, JNK activation, nuclear translocation of forkhead BOX O1 (FOXO1) [128], as well as apoptotic signals, ER stress, and oxidative stress [129].

### 7.2 Cardiovascular Diseases

The heart has higher levels of metabolic activity. The myocytesdepends on mitochondria for energy production and is, therefore, highly prone to ROS production. According to studies, oxidative stress is a major factor in the onset and advancement of cardiovascular disorders, particularly coronary artery disease (CAD). CAD causes both acute and chronic episodes of myocardial ischemia, resulting in oxidative stress in cardiomyocytes, which affects myocardial contractility and ultimately leads to cardiac failure [130]. Therefore, ROS plays the main role in normal cell signaling pathways and cardiovascular physiology.

Cardiac pathologies can disrupt calcium homeostasis or cellular redox status, leading to protein misfolding and the development of proteotoxic soluble peptides. This activates HSPs to repair protein misfolding and protect the heart muscle from oxidative stress [131]. HSPs confer cardio protection by enhancing cellular resistance to oxidative stress and hypoxia, leading to increased functional recovery and reduced infarct size following experimental ischemia [132]. Recent studies have highlighted HSP90 as a viable therapeutic target for ischemia injury. In non‐re‐perfused myocardial ischemia models, pretreatment with HSP90 inhibitors during cardioplegia has been shown to decrease infarct size, fibrosis, and macrophage infiltration [133]. HSP70 also demonstrates strong cardioprotective effects. HSP70 and p‐p38 MAPK (p38 mitogen‐activated protein kinase) levels significantly increased in rat cardiomyocytes after they were subjected to oxygen‐glucose deprivation and reperfusion [134]. Similar findings were reported in myocardial injury induced by ischemia, where suppressing HSP70 activity with quercetin resulted in a notable enlargement of the infarcted area. Furthermore, the inhibition of HSP70 was associated with elevated levels of Phosphorylated p38MAPK and STAT3 (P‐STAT3), alongside a reduction in SERCA2 expression during the ischemic injury [135]. HSP22 possesses a key function in cardiac adaptation to oxidative stress. Its expression is significantly elevated under cardiac stress situations, and overexpression shields the heart from the ischemic injury by promoting the production of iNOS [136]. Apart from inhibiting ROS, HSP27 interacts with Akt, maintaining its stability, thereby downregulating the TNF‐α and IL‐1β mRNA levels [137]. Studies on isolated rat heart in a Langendorff system have demonstrated that elevated HSP20 expression protects against ischemic injury. This protective effect is evidenced by full restoration of heart function, lower infarct size, and reduced myocardial cell death, which occurs through modulation of the Bcl2/Bax balance and suppression of caspase‐3 activity. These results highlight the role of HSP20 as an effective alternative for treating ischemic heart disease [138].

### 7.3 Autoimmune Diseases

Autoimmune diseases are described by an inappropriate immune response directed against self‐antigens, resulting in chronic inflammation and tissue damage. Among the many contributing elements, ROS is an important factor in the development of autoimmune disorders. ROS contributes to the pathophysiology of autoimmune disorders by aggravating immunological dysregulation via the promotion of oxidative stress. ROS disrupts immune regulation, which causes inflammatory signaling pathways to be activated and neo‐antigens to develop. This further promotes tissue damage by triggering the production of pro‐inflammatory cytokines and autoantibodies [139]. HSPs are essential for controlling autoimmune disorders. HSPs can modulate ROS‐induced immune dysfunction by reducing oxidative stress, suppressing inflammatory responses, and stabilizing cellular proteins. Although HSPs are highly immunogenic, the way they affect autoimmune disorders varies based on the particular protein and their cause. Several investigations have looked at the possibility of HSPs as a treatment for autoimmune diseases. For instance, hypersensitivity to the HSP60 chaperone has been shown to restrict immune responses in animal models of rheumatoid arthritis (RA) and juvenile idiopathic arthritis (JIA) [140]. Clinical trials have also shown promising results. In a study, individuals with early‐stage RA were given a highly conserved bacterial HSP‐40‐derived peptide (dnajp1). The results showed that tolerance to the therapy increased levels of regulatory cytokines, including IL‐4 and IL‐10, whereas T‐cell proliferation and the production of pro‐inflammatory cytokines decreased [141]. HSP70 and HSP90 are two significant HSPs that are frequently linked to autoimmune diseases. In a variety of situations, HSP70s have an immunoregulatory role that lowers inflammation and promotes the development of regulatory T cells (Tregs) [142]. For example, HSP70 therapy induced Treg cell expansion and increased lymphocyte‐activation Gene 3 (LAG3) expression, leading to a reduction in disease impact, in a mouse model of autoimmune arthritis. Additionally, HSP70‐histone H1 linker protein 1 (HSP70‐HINT1) downregulated immune responses in a study autoimmune encephalomyelitis model. Additionally, HSP90 has been successfully shown to reduce autoimmunity in mice with Type 1 diabetes, experimental autoimmune encephalomyelitis, and skin disorders [143]. Moreover, in autoimmune arthritis rats, HSP90 was shown to alleviate disease symptoms and promote immune tolerance [144].

### 7.4 Neuronal Disorders

In neurological disorders including Parkinson's, and Alzheimer's illness, excessive ROS and oxidative stress often cause neuronal damage and cell death. The brain regions that are most vulnerable to oxidative stress include prefrontal cortex, amygdala, cerebellar granular cells and hippocampus [145]. Neurons and other cellular elements of the central nervous system have been found shown to undergo damage from oxidative stress. For instance, astrocytes contribute to appropriate inflammatory responses by producing elevated ROS. However, excessive oxidative stress can lead to astrocyte‐associated inflammation and concomitant astrogliosis [146, 147].

The overproduction of ROS has been linked to mitochondrial dysfunction, which disrupts energy generation, alters metal ion balance and promotes formation of harmful proteins clumps, all of which are hallmarks of several neurodegenerative disorders. In astrocytes, ROS has showed to activate several inflammatory signaling pathways, resulting in inflammatory mediators release and often causing astrogliosis. ROS triggers astrocytic‐NLRP3 inflammatory cascades through triggering procaspase‐1. Capase‐1 releases IL‐1β and IL‐18. Increased release of these pro‐inflammatory cytokines (IL‐1β and IL‐18) can make neuronal damage [148]. H_2_O_2_ promotes ROS‐driven apoptosis in astrocytes by triggering NF‐κB signaling pathway. This oxidative stress response causes astrocytes to secrete proinflammatory cytokines, through their close communication with microglia, initiate microglial activation and intensify the neuroinflammatory cascade [149].

HSPs can rapidly increase their expression in acute brain injuries (such as ischemia). For instance, the levels of HSP B1 (HSPB1) are much higher in astrocyte cells and lower in specific neurons of the ischemic brain tissue during middle cerebral artery blockage. Under stress, HSPB1 binds to Glial Fibrillary Acidic Protein to maintain the astrocyte cytoskeleton [150]. Another study showed that HSPB5 and HSPB1 are highly expressed after ischemia/reperfusion injury, primarily in neurons of the infarcted cortex compared to glial cells. Phosphorylation of HSPB1 and HSPB5 has been shown to occur after ischemia, suggesting that it may influence their neuroprotective role. Amyloid‐beta (Aβ) can impair multiple aspects of mitochondrial function, including the generation of ROS, energy metabolism dysfunction, and others [151]. HSP90 and HSP70 have a crucial role in regulating Tau protein phosphorylation and aggregation. By disrupting fibrillogenesis, Aβ amyloid peptide aggregation can be inhibited by HSP60. Even though HSP60 plays neuroprotective function in AD, further study is required to be clarifying the mechanisms of action and therapeutic applications [44].

### 7.5 Respiratory Disorders

Cigarette smoke and exposure to air pollutants like ozone, NO₂, and SO₂ can cause direct injury to lung tissue. These irritants also stimulate the generation ROS, contributing to inflammatory responses within the lungs. Excessive ROS production causes oxidative stress, which increases inflammation, apoptosis, and tissue damage in the lungs [152]. However, elevated ROS levels directly oxidize the lipids, protein, and DNA, which leads to lung damage the cellular responses via the production of ROS. ROS possess the potential to modify several aspects of lung function, including ECM remodeling, immunological regulation, surfactant and antiprotease screen maintenance, cell proliferation, apoptosis, and mitochondrial respiration [153]. Moreover, elevated ROS are linked to the start of the lung inflammatory response by activating transcription factors like AP‐1 (activator protein‐1) and NF‐κB, signaling MAP kinase pathways, PI3K (phosphoinositide 3‐kinase), PI‐3K‐activated serine kinase AKT, and histone acetylation/deacetylation (chromatin modeling), which leads to the expression of proinflammatory mediators at the gene level. ROS production by phagocytes that have been driven to inflammatory areas is thought to be a primary factor in the cell damage linked to several persistent inflammatory lung disorders such as COPD and asthma [154].

HSPs, notably HSP27, HSP70, and HSP90, combat ROS activity by combining antioxidant defense, protein protection, and control of inflammatory and apoptotic pathways. HSPs, such as HSP70 and HSP90, role as molecular chaperones, confirming normal protein folding, misfolded proteins and refolding, limiting the harmful buildup of protein aggregates in lung tissues [54]. HSP70 and HSP90 decrease pro‐inflammatory cytokines including TNF‐α and IL‐6 by suppressing NF‐κB activation. This modulates the inflammatory reaction. This function helps to decrease excessive inflammation and tissue injury in disorders such as asthma and ARDS [155]. HSP70 expression is often increased as a compensatory strategy to combat the oxidative damage caused by cigarette smoke. Studies have revealed that increasing HSP70 expression might minimize ROS‐mediated lung damage and suppress inflammation, giving a possible therapeutic strategy [62]. The antioxidant and cytoskeletal stabilizing properties of HSP27 aid in preventing ROS‐induced damage to airway cells, enhancing lung function, and lessening the intensity of asthma symptoms [154].

## 8 HSP's Pathogological Mechanisms

HSPs can also contribute to the development of disorders such as diabetes, neurological diseases, and respiratory diseases by triggering autoimmune responses, they exhibit a dual role both protective and pathogenic.

Cells can enhance HSPs in response to various external and internal stresses as a protective mechanism. However, when cells become severely injured or undergo necrosis, intracellular HSPs may be released into the extracellular space. This release can occur through passive leakage from damaged or necrotic cells, active secretion via extracellular vesicles, or presentation to T lymphocytes by antigen‐presenting cells through major histocompatibility complex (MHC) molecules. HSPs presence in extracellular spaces develops autoimmunity because HSPs have pro and anti‐inflammatory effects. Determining the significance of extracellular HSPs in immune response is also confounded by the fact that increased titers of autoantibodies against HSPs are often observed in patients suffering from various inflammatory diseases [156, 157]. The interaction of extracellular HSPs with immune cells may stimulate humoral (auto) immune response and lead to the production of anti‐HSP (auto) antibodies, which are elevated in many inflammatory and autoimmune diseases.

Several findings confirm that autoantibodies against HSPs are present in selected autoimmune diseases. Increased anti‐Hdj2 (HSP40) IgG and anti‐Hdj3 (HSP40) IgG levels are associated with serum levels of IL‐6, positive correlation between serum levels of anti‐HSP60 IgG and IL‐4, and positive correlation between serum levels of anti‐HSP90 IgG and IFN‐γ are presented in RA [158]. In JIA, higher level in circulation positive correlation between serum levels anti‐HSP70 IgG/M and disease severity has been founded. Anti‐HSP40/60/90 IgG higher levels have been recognized in circulation and positive correlation between serum levels autoantibodies against tissue transglutaminase [159]. Passive transfer of anti‐HSP70 IgG led to attenuation of disease activity and inhibition of the proinflammatory Th17 population in a psoriasis mouse model. Anti‐HSP90α IgG can increase psoriasis disease severity [159]. Therefore, HSPs can also contribute to the development of disorders such as diabetes, neurological diseases, and respiratory diseases by triggering autoimmune responses, they exhibit a dual role both protective and pathogenic.

In some cases, therapeutic challenges are present. For instance, certain HSPs show dual actions. HSP70 acts as an immunosuppressant intracellular by inhibiting activity of NF‐κB. In contrast, HSP90 develops the inflammation by promoting activity of signaling factors like JAK‐STAT and transcriptional factor NF‐κB. Pharmacological inhibition of HSP90 activity leads to silencing of the inflammatory response, which has been proven in many clinical studies. Thus, these kinds of challenges can be sorted out for instance, combination of HSP90 Inhibitors and HSP70 Inducers Prevent Hydrochloric Acid‐Induced Pulmonary Fibrosis in Rabbits [160]. HSP90 inhibitors and combined HSP90 inhibitors and HSP70 inducers inhibit ERK activation (phosphorylation) in mice [161]. Recent studies show that HSPs are deeply involved in the pathogenesis of fibrotic diseases across multiple organs, including the kidney, liver, lung, myocardium, pancreas, skin, peritoneum, and intestine. Among them, HSP110 and HSP70 consistently exhibit anti‐fibrotic functions, with evidence indicating that HSP110 can suppress myocardial hypertrophy, while HSP70 serves as a classic fibrosis‐inhibiting chaperone. In contrast, HSP90, HSP47, and HSP27 promote fibrotic processes and are associated with tissue remodeling and ECM deposition. Although the field is still evolving, these findings highlight the diverse and context‐dependent roles of HSPs in fibrotic disease progression [162]. HSP expression is linked to ischemia‐reperfusion damage and inflammatory symptoms after abdominal organ transplants. This may affect the graft outcome and is a prognostic factor [163].

## 9 Therapeutic Strategies and Pharmacological Targeting of HSPs in Various Disorders

HSPs have emerged as interesting targets for treating various disorders. Their involvement in enhancing cell survival has made them a target for therapeutic studies. HSP70 overexpression suppress anti‐apoptotic proteins including Bcl‐2 and Bcl‐xL and stops JNK‐induced phosphorylation. It also inhibits the apoptosis inducing and apoptotic protease activating factor. This mechanism helps maintain mitochondrial stability and ultimately protects cells from apoptosis. In contrast, HSP70 levels have been shown to be low in a variety of different human disorders, including pulmonary fibrosis, myopathies, cholesterol sphingolipidosis, diabetes, and obesity [164]. HSP72 inhibits inflammation in cells by lowering pro‐inflammatory cytokines levels including IL‐6, TNF‐α, IL‐1β while HS70 regulates in blood vessels [144]. HSP60 performs a variety of functions, including stress regulation, mitochondrial integrity maintenance, immunological response, and pro‐ and anti‐apoptotic actions. For instance, HSP60 suppresses NF‐κB kinases activation, that has a protective action towards the cells from the oxidation stress generated via NF‐κB‐targeted gene regulation. In addition, HSP60 class of proteins are associated with human disorders that include cardiovascular disease, diabetes (Type 2), JIA, hepatitis B, atherosclerosis, and various cancers [165, 166, 167]. HSP27 has emerged as a pivotal player in neurodegenerative diseases, particularly PD. Its role in modulating glycation‐associated cellular pathologies and its association with α‐synuclein make it a promising therapeutic target and biomarker. HSP27's ability to bind and inhibit amyloid nucleation, fibril binding, and fibril disaggregation highlights its role in preventing the harmful effects of glycation‐induced protein aggregation. HSP27 represents a promising therapeutic target for PD. Strategies aimed at enhancing HSP27 expression or activity could potentially delay disease progression. Furthermore, HSP27 in neuronal cells plays a key role by mitigating the damage caused by oxidative stress and protein misfolding [168].

HSPs are being explored as possible targets for reducing the negative effects in some disorders by increasing their levels. Resveratrol stimulates the SIRT1 (Sirtuin1) signaling pathway, which increases HSPs levels (HSP70, HSP90, and HSP60). This activation improves antioxidant enzyme activity and tight junction protein expression, providing protection against heat stress‐induced inflammation. Resveratrol has also been found to avert the NLRP3/NF‐κB inflammasome signaling pathway, reducing the secretion of IL‐1β. This implies that HSPs have a vital role in regulating inflammatory interactions [169]. Geranylgeranyl Acetone (GGA), an isoprenoid compound, induces the synthesis of HSPs (HSP70, HSP90, HSP105) in several tissues, including the central nervous system, liver, retina, and heart. This suggests its potential therapeutic applications in conditions where HSPs are beneficial [170]. Curcumin, myricetin, cisplatin, phencyclidine, and sulforaphane (SFN) have also been shown to elevate HSP70 and HSP72 expression. SFN induces a rapid and significant Hsf1‐mediated heat shock response, leading to increased HSP27 expression [171]. However, HSPs can be pharmacologically activated or suppressed for therapeutic gain, and many natural or synthetic compounds function as HSP inducers or inhibitors.

Curcumin, a polyphenolic compound from *Curcuma longa*, is a well‐known HSP inducer. It enhances HSP27 and HSP70 expression under proteotoxic stress, both in vitro (rat glioma cells, rat hepatocytes, and mouse fibroblasts) and in vivo (heat‐stressed rats). This effect is mediated through the formation of an intermediate, activated form of HSF1. Paeoniflorin, isolated from *Paeonia lactiflora* and the fern *Salvinia molesta*, also induces HSP expression by activating HSF1 and improves thermotolerance in mammalian cell models. Glycyrrhizin, a major constituent of liquorice root, similarly promotes HSP expression and shows synergistic effects when combined with other heat‐shock activators [172]. Geldanamycin, a benzoquinone ansamycin antibiotic from *Streptomyces hygroscopicus*, induces HSP40, HSP70, and HSP90 through interactions with huntingtin exon 1 protein. Although geldanamycin and its derivatives can activate small HSPs and stimulate the heat‐shock response in normal tissues, they also inhibit Hsp90 in cancer cells an action that undesirably increases compensatory HSP70 and HSP90 expression. Their therapeutic use is further limited by poor blood–brain barrier penetration and destabilization of essential growth‐factor receptors and signaling proteins, despite their potential neuroprotective properties [173]. The novobiocin derivative KU‐32 modulates Hsp90 function by binding to its C‐terminal domain, promoting a partially closed N‐terminal conformation that enhances ATPase activity. Bimoclomol, another small molecule, binds to the HSF1 complex and amplifies HSF1–DNA binding, thereby augmenting the heat‐shock response [174]. In contrast, HSP inhibition particularly targets HSF1, the central transcriptional regulator of the proteotoxic stress response has emerged as a promising anticancer strategy. HSF1 supports cancer cell survival by enabling adaptation to oncogenic and environmental stress, making it a more attractive therapeutic target than HSP90 or other individual HSPs.

The first HSF1 inhibitor identified from nature was quercetin, a simple flavonoid. Stresgenin B, isolated from *Streptomyces* sp. AS‐9, exhibits similar activity by suppressing heat‐induced HSP gene expression. Triptolide, a diterpene triepoxide, is another potent natural HSF1 inhibitor. Synthetic inhibitors such as KRIBB11 and IHSF115 directly bind to HSF1 and interfere with its transcriptional function, whereas most newer compounds act indirectly by blocking HSF1 activation pathways [175]. Recent experimental and clinical studies on HSPs have highlighted several promising therapeutic directions. For example, the resorcinol‐based HSP90 inhibitor AT1387 was recently identified as capable of preserving the alveolo‐capillary barrier and preventing HCl‐induced chronic lung injury and pulmonary fibrosis when administered at low, non‐toxic doses. In addition, ganetespib (formerly STA‐9090), a highly potent and selective HSP90 inhibitor featuring a resorcinol moiety and a distinctive triazolone structure, is currently being tested across multiple clinical trials [44].

## 10 Future Perspectives and Conclusion

The complex relationship between stress, ROS, and HSPs at the cellular level has attracted the attention of researchers, particularly when they are influenced by diverse external stressors such as food, alcohol, heavy metal, heat, radiation exposure, and intracellular stressors are such as inflammatory cytokines, autoimmune diseases, obesity, and neuroinflammatory disorders etc. As research in this area continues, numerous fascinating possibilities for further investigation emerge. While Future research might concentrate on understanding the individual signaling mechanisms that allow the ROS‐HSP interaction, notably how HSPs may selectively activate or decrease ROS production depending on the kind of external stress and internal stressors. The link between HSPs, stress, and ROS is an intricate and dynamic area of study with broad implications for comprehending CS responses and developing novel biotechnological and medicinal applications. Further research will need to focus on the context‐specific roles of these proteins and molecules, as well as their ability for aimed modulation in health and disease. As our understanding of these interactions develops, new possibilities for averting the negative consequences of stress at the cellular level will likely emerge, opening the way for novel approaches to improving cellular resilience and stress tolerance in a variety of circumstances. Since HSPs exhibit tissue‐dependent actions, with anti‐fibrotic functions in myocardial hypertrophy (HSP70, HSP110) and pro‐fibrotic roles in others (HSP27, HSP47, HSP90), future studies must dissect these context‐specific mechanisms across organ systems such as the lung, liver, kidney, myocardium, pancreas, and skin. Comparative analyses using single‐cell models, conditional knockout animals, and organoid systems could help identify how local microenvironment, cell type, or disease stage determines the functional polarity of each HSPs. This will aid in predicting therapeutic outcomes and avoiding unintended exacerbation of fibrosis or inflammation. Thus, the synergistic benefits observed with combined HSP70 inducers and HSP90 inhibitors (e.g., in hydrochloric acid–induced pulmonary fibrosis and ERK inhibition models) highlight the promise of combination strategies. Future work should evaluate optimal dosing, timing, and sequence of HSPs co‐modulation, as well as their interactions with current anti‐inflammatory, anti‐fibrotic, and immunomodulatory drugs.

To comprehensively understand the regulatory networks governed by HSPs, future research should integrate multi‐omics approaches including genomics, transcriptomics, proteomics, phosphoproteomics, and metabolomics. Systems‐level analysis will help unravel how HSPs influence key pathways such as NF‐κB, JAK‐STAT, ERK, and TGF‐β signaling under different stress conditions. Omics‐guided biomarker discovery could also identify patient subgroups that may respond to HSPs‐based therapies, supporting precision medicine strategies for inflammatory and fibrotic diseases. Although several preclinical models support the therapeutic potential of HSPs modulators, clinical translation is needed to bring the benefits to the therapeutic arenas. This includes validation of safety profiles, pharmacokinetics, long‐term outcomes, and identification of reliable surrogate markers of HSPs activity. Large‐scale trials should explore whether personalized HSPs modulation can attenuate fibrosis progression additionally, inflammatory responses in human diseases and organ transplants.

## Author Contributions

Conceptualization: Paka Sravan Kumar and Anoop Kishore. Supervision: Anoop Kishore. Writing – Original draft: Paka Sravan Kumar and Anoop Kishore. Review and editing: Triveni Kodi, Adarsh Gopinathan, and Krishnadas Nandakumar. Image visualization: Bharath H B.

## Conflicts of Interest

The authors declare no conflicts of interest.

## Data Availability

Data sharing is not applicable to this article as no datasets were generated or analyzed during the current study.
